# Earth Abundant Element Type I Clathrate Phases

**DOI:** 10.3390/ma9090714

**Published:** 2016-08-23

**Authors:** Susan M. Kauzlarich, Fan Sui, Christopher J. Perez

**Affiliations:** Department of Chemistry, One Shields Ave, University of California, Davis, CA 95616, USA; fsui_ucd@hotmail.com (F.S.); cpe@ucdavis.edu (C.J.P.)

**Keywords:** silicon, photovoltaics, thermoelectrics

## Abstract

Earth abundant element clathrate phases are of interest for a number of applications ranging from photovoltaics to thermoelectrics. Silicon-containing type I clathrate is a framework structure with the stoichiometry A_8-x_Si_46_ (A = guest atom such as alkali metal) that can be tuned by alloying and doping with other elements. The type I clathrate framework can be described as being composed of two types of polyhedral cages made up of tetrahedrally coordinated Si: pentagonal dodecahedra with 20 atoms and tetrakaidecahedra with 24 atoms in the ratio of 2:6. The cation sites, A, are found in the center of each polyhedral cage. This review focuses on the newest discoveries in the group 13-silicon type I clathrate family: A_8_E_8_Si_38_ (A = alkali metal; E = Al, Ga) and their properties. Possible approaches to new phases based on earth abundant elements and their potential applications will be discussed.

## 1. Introduction

Inorganic clathrate phases which can be described as all-inorganic frameworks with guest atoms have been of interest for a wide variety of applications, including thermoelectrics, Li-ion batteries, hydrogen storage, and photovoltaics [1]. In particular, silicon (Si)-containing clathrate phases are of significant interest because of being earth abundant and environmentally benign. The alkaline earth-containing group 13-Si light element clathrates have been reviewed recently [2]; therefore, this short review provides an update on the alkali metal-containing group 13-Si compounds crystallizing in the clathrate type I structure.

**Figure 1:**
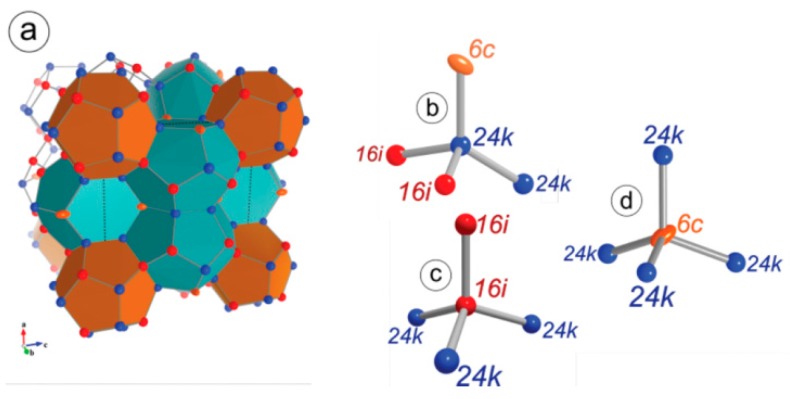
(**a**) Crystal structure of type I clathrate framework. Local geometry and connectivity of framework sites (**b**) 6*c*; (**c**) 16*i*; (**d**) 24*k*. (Reprinted with permission from Roudebush, J.H.; de la Cruz, C.; Chakoumakos, B.C.; Kauzlarich, S.M. Neutron Diffraction Study of the Type I Clathrate Ba_8_Al_x_Si_46-x_: Site Occupancies, Cage Volumes, and the Interaction between the Guest and the Host Framework. *Inorg. Chem.* **2012**, *51*, 1805–1812 [6]. Copyright (2012) American Chemical Society.

Na_8-x_Si_46_ is the first observed silicon-based compound discovered to crystalize in the type I clathrate structure. The structure can be described as having a covalently bonded silicon (Si) framework with sodium (Na) as the guest atom centered within the polyhedra [3]. The structure has been described in detail many times and consists of Na cations encapsulated in tetrahedrally bonded silicon framework (cubic space group, Pm-3n), shown in Figure 1. The framework contains 46 atoms with three unique sites typically referred to by their Wyckoff positions 6*c*, 16*i*, and 24*k*. The eight guest atoms in the unit cell are located at the Wyckoff positions 2*a* and 6*d*, which centers the two types of polyhedra found in type I clathrate: 20-vertex pentagonal dodecahedra and 24-vertex tetrakaidecahedra. Following the discovery of Na_8-x_Si_46_, the alkali metals series (A = K, Rb, Cs) of type I clathrate were synthesized from the elements by annealing, with the Cs clathrate being a high pressure phase [4,5]. The alkali metals´ guest atoms in the silicon-containing clathrate phases donate electrons, and these phases are metallic [1].

Clathrate phases with the composition A_8_E_8_Si_38_ (A = alkali metal, E = Al, Ga, In) are considered to be electron-balanced phases as the compounds contain an equivalent amount of group 13 elements to accept the donated electrons from guest atoms and were predicted to show semiconducting behavior [7,8]. The phases prepared to date are A_8_E_8_Si_38_ (A = Na [9,10], K [7,10,11,12], Rb [8], Cs) [13,14] and have been shown experimentally to be semiconducting when the composition is near the expected; the E = In and A = Na, E = Ga phases have not been prepared. Table 1 provides the lattice parameters of the A_8_E_8_Si_38_ compounds reported to date. The only borosilicide compound with type I clathrate structure is K_7_B_7_Si_39_ [15], although boron has been incorporated into a type I Ba_8_Al_16_Si_30_ clathrate at dopant levels [16]. Besides group 13 substitutions in the Si framework, silicon clathrates containing earth abundant transition metal substitutions A_8_M^II^_4_Si_42_ have been prepared with M^II^ = Zn: K_8_Zn_3.5_Si_42.5_ and Rb_7.9_Zn_3.6_Si_42.4_ [17]. While the heavier group 14 elements are not a topic of this review, it is noteworthy that, while most of the compositions described are n-type semiconductors, there is at least one example of a p-type compound with Sn, K_8_Ga_8.5_Sn_37.5_ [18]. Since thermoelectric devices need both n- and p-type semiconductors, the possibility that both can be prepared provides additional incentive for developing the chemistry of the silicon clathrates further. Type II clathrate phases composed of group 13 elements and Si have also been prepared, Cs_8_Na_16_Ga_23_Si_113_, Rb_8.4_Na_15.6_Ga_20_Si_116_ [19], and Cs_8_Na_16_Al_24_Si_112_ [20], demonstrating new horizons for further development of alkali metal-containing clathrates. Aside from structure, only the thermoelectric transport properties of the type II clathrate Cs_8_Na_16_Al_24_Si_112_ have been reported to date [20].

## 2. Theory

Interest in these electron-precise clathrate structures, A_8_E_8_Si_38_, has resulted in theoretical investigations of their formation energies, bandgaps and other transport properties [11,21,22,23]. These silicon-containing clathrates are of interest for both photovoltaics and thermoelectrics. The interest in photovoltaics is a result of the possibility of a direct bandgap in the appropriate range for solar energy conversion [11,12,13]. Even without being direct gap semiconductors, the idea that charge recombination can be reduced or minimized has led to a resurgence of interest [22]. Clathrate structures for thermoelectric applications have been considered ideal Phonon Glass Electron Crystal (PGEC) materials [24]. PGEC has been an important concept for designing efficient thermoelectric materials [23]. The idea is that a material can have properties similar to a glass for phonons and as a crystal for electrical transport. This allows for low thermal conductivity and high electrical conductivity: two important parameters for efficient thermoelectric materials [23]. Silicon and earth abundant clathrate phases have been particularly desirable as possible PGECs, as many of the most efficient materials for direct heat to electrical conversion contain critical or environmentally unfriendly elements.

In general, clathrate structures form a cage network that encapsulates an element referred to as the guest. The optimal material for thermoelectric applications would be where the guest atom is weakly bound to the cage and provides a vibrational mode referred to as a “rattling mode” that can scatter the lattice phonon mode. While reduction in thermal conductivity in these type I clathrate phases has been attributed to the guest vibrational modes, first principle calculations also demonstrate that structural disorder of the covalently bonded cages plays an important role in reducing the thermal conductivity [11,22].

**Figure 2:**
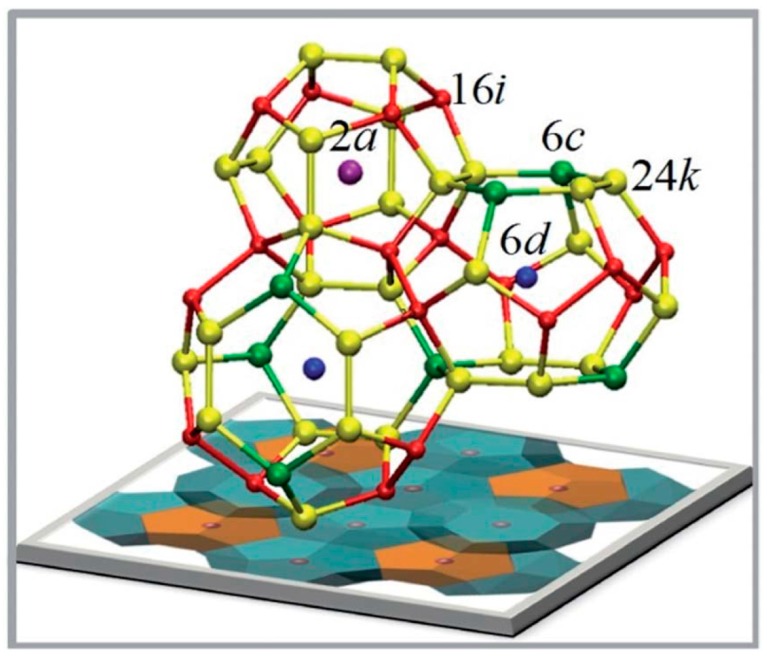
A view of the type I clathrate structure with the Wyckoff site symbols indicated. The network structure consists of 6*c* (green), 16*i* (red), and 24*k* (yellow) and the two guest sites are denoted by 2*a* (magenta) and 6*d* (blue). Reproduced from He, Y.; Sui, F.; Kauzlarich, S.M.; Galli, G. Si-based Earth abundant clathrates for solar energy conversion. *Energy Environ. Sci.* **2014**, *7*, 2598–2602 [11] with permission from The Royal Society of Chemistry.

The band structures of the series A_8_E_8_Si_38_ (A = Na, K, Rb, Cs; E = Al, Ga, In) were calculated using density functional theory [25,26]. The calculations indicate that the bandgap remained indirect, but became larger with the heavier guest alkali atom with an estimated gap of ~0.9 eV for A = Na and ~1.36 eV for A = Cs. The calculations indicate that the bandgap was not very sensitive to the group 13 element, E, but again followed the principle of becoming slightly larger with heavier elements. The authors noted that the theoretical predictions might not be valid because of site-specific positioning of the atoms, vacancies, or deviations from ideal stoichiometry, but they are fairly close to the experimental values. Additional theoretical calculations on the series, focusing on the group 13 site preferences, was reported [21]. Site specificity of the group 13 element within the clathrate I framework has been noted experimentally, so detailed calculations are of great interest. As mentioned above, there are three framework sites that the group 13 element could occupy. In a simplistic view, the group 13 element occupies the site most likely to avoid any E-E bonding interactions in order to better satisfy valence [27]. Figure 2 shows how the specific sites that will be discussed further are interconnected with the Wyckoff nomenclature indicated. In these type I clathrates, the group 13 element favors the 6*c* framework site with a small amount on the 24*k* site in the ratio of approximately 5:3, when A = K, Rb, Cs. This is consistent with experimental observations for these alkali metal clathrates [7,8,9,11,13].

Theoretical calculations suggest that these phases should have applications in photovoltaics with K_8_Al_8_Si_38_ showing a quasi-direct bandgap of ~ 1 eV with relatively high mobilities determined [22]. Additionally, investigation of the valence band and conduction band-wave functions revealed clear distinction in spatial location, thereby suggesting low probability of charge carrier recombination and efficient energy conversion. The transport properties related to thermoelectrics have also been calculated for the E = Ga phases and efficiencies predicted with the best energy conversion phase being hole-doped Cs_8_Ga_8_Si_38_ [21]. Overall, there has been significant progress in both theoretical and experimental investigations suggesting that fundamental understanding of the physics and chemistry will be advanced along with the practical applications of these materials [23].

## 3. Structure

Powder X-ray diffraction was performed on the binary type I clathrate phases K_7.62(1)_Si_46_ and Rb_6.15(2)_Si_46_ to examine their crystal structure [4]. The host atom sites have no vacancies revealed by Rietveld refinement of the X-ray powder diffraction pattern; the Si frameworks are fully occupied similar with that of Na_8_Si_46_ [28]. Fully occupied framework sites are commonly noticed in alkali metals-Si clathrate phases; in the contrast, Ge or Sn clathrates with alkali metals have been reported to possess vacancies at the framework’s 6*c* site [8,29,30,31,32,33]. Ramachandran et al. attributed the difference in the framework vacancy formation to the weaker homoatomic bonding descending the group in the periodic table [4].

Compounds with the stoichiometry A_8_E_8_Si_38_, A = Na, K, R, Cs and E = Al and Ga have been prepared to date and their lattice parameters are provided in Table 1. In ternary clathrate phases, the substitution preference of silicon framework sites (6*c*, 16*i*, 24*k*) can affect the band structure and transport properties significantly. In the A_8_E_8_Si_38_ framework, Ga occupancy can be distinguished by single-crystal X-ray diffraction as Ga and Si atoms have electron densities that provide significantly different scattering factors. For A = K, Rb, Cs compounds, the Ga occupancy ratio at the 6*c* site is in the range of 61%~46% and 17%~20% at the 24*k* site. At the 16*i* site, the Ga occupancy ratio is low, only 1%~2%, which indicates Ga preference for the 6*c* site (Table 2) [13]. Similarly, Zn also shows site preference for the 6*c* site and avoids substitution on the 16*i* site [17]. Distinguishing Al from Si in A_8_Al_8_Si_38_ (A = K, Rb, Cs) is difficult, so neutron powder diffraction was performed to study the Al/Si mixed occupancy ratios [14]. Even with neutron diffraction, it is difficult to distinguish Al from Si. Rietveld refinement of the neutron powder diffraction gave the total chemical formula of A/Al/Si close to the nominal stoichiometry 8/8/38. Al’s site occupancy fraction (*s.o.f.*) is shown in Table 2 to compare with the Ga’s *s.o.f.* The group 13 atoms’ preference to occupy 6*c* site is attributed to the fact that there is no direct bonding between 6*c* sites (shown in Figure 2), and group 13 atoms occupy these specific sites in order to avoid the disfavored 13-13 bonds [27]. Consistent with what is found in the A_8_Ga_8_Si_38_ samples, Al prefers 6*c* site and avoids 16*i*. Additionally, as the unit cell expands with larger guest atoms from K to Cs, the *s.o.f.* of Al at the 6*c* site decreases. This is similar to what has been discovered in Ba_8_Al_x_Si_46-x_ samples by neutron diffraction study; when *x* increases from 6 to 15, to accommodate more Al atoms, more Al atoms go to 24*k* site [6,34].

The theoretical calculations discussed above also identified the two most stable configurations as being consistent with what is found experimentally: 6 Al atoms at 6*c* and 2 Al atoms at 16*i*, or 5 Al atoms at 6*c*, 2 Al atoms at 24*k* and 1 Al atom at 16*i*.

**Figure 3:**
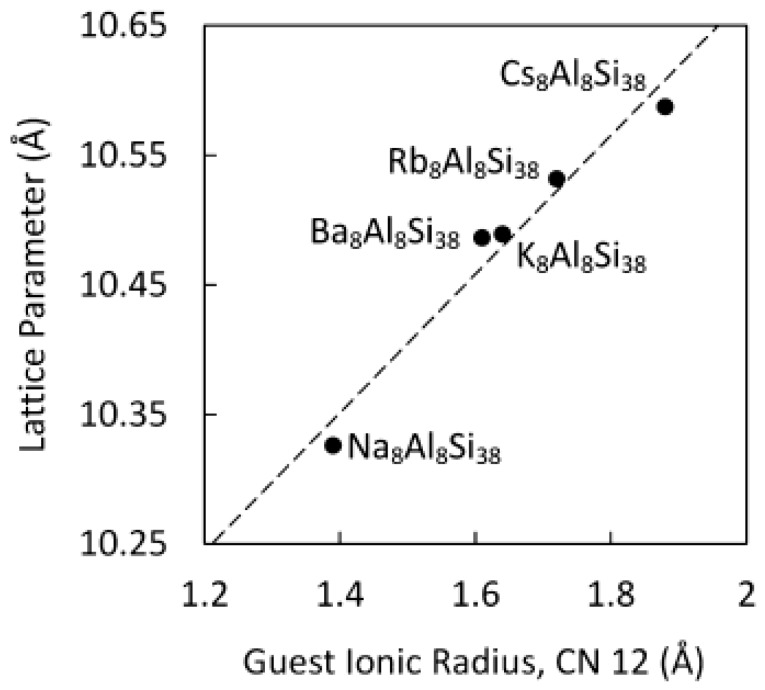
Lattice parameter vs. guest ionic radii for coordination number (CN) = 12 for A_8_Al_8_Si_38_. Reprinted with permission from Dong, Y.K.; Chai, P.; Beekman, M.; Zeng, X.Y.; Tritt, T.M.; Nolas, G.S. Precursor Routes to Complex Ternary Intermetallics: Single-Crystal and Microcrystalline Preparation of Clathrate-I Na_8_Al_8_Si_38_ from NaSi plus NaAlSi. *Inorg. Chem.* **2015**, *54*, 5316–5321 [9]. Copyright (2015) American Chemical Society.

Na_8_Al_8_Si_38_ has only been prepared by kinetically controlled thermal decomposition (KCTD) of a mixture of NaSi [35] and NaAlSi [36]. This phase cannot be prepared by conventional synthetic routes so may be a metastable or kinetically stable phase. Small single crystals and phase-pure sample were obtained. The lattice parameter vs. unit cell volume, shown in Figure 3, is in good agreement with that expected for A = Na when compared to A_8_Al_8_Si_38_ (A = K, Rb, Cs). Rietveld and single-crystal refinements indicate that Al preferentially resides at the 6*c* site in the ratio Al:Si 0.79:0.21. The 24*k* site to a significantly lesser extent with the ratio Al:Si 0.13:0.87 and no evidence for Al on the 16*i* site. This result is similar to what has been determined for A_8_Al_8_Si_38_.

In the case of K_7_B_7_Si_39_, where B is a much smaller atom than Si, B is found to substitute 16*i* sites by single-crystal X-ray diffraction refinement, while only Si is found at 6*c* and 24*k* sites [15]. This is very different than that observed for A_8_E_8_Si_38_ (E = Al, Ga). In Figure 2, it can be seen that the six-membered ring of the 24-vertex tetrakaidecahedral cage, composed of 6*c* and 24*k* sites, has bond angles deviating from an ideal tetrahedron and therefore creates chemical stress if substituted by the small B atom. Therefore, B atoms have a preference for the 16*i* site [15].

In the type I clathrate crystal structure, the guest atom Wyckoff nomenclature is 2*a* inside the 20-vertex pentagonal dodecahedral cages and 6*d* inside the 24-vertex tetrakaidecahedral cages. Large anisotropic atomic displacement parameters (ADPs) have been reported for the 6*d* sites guest atoms, and therefore they are considered as rattler and contribute to phonon scattering. For compounds K_7.62(1)_Si_46_ and Rb_6.15(2)_Si_46_, when guest atom vacancy is present, occupancy at 6*d* site is preferred and 2*a* site has lower *s.o.f*, 88.5(4)% at K_7.62(1)_Si_46_ 2*a* site and 21.8(5)% at Rb_6.15(2)_Si_46_ 2*a* site [4]. Similarly, in sample Cs_7.8(1)_Si_46_, *s.o.f* of 2*a* site is 0.881(4) while 6*d* site is close to fully occupied [5].

**Figure 4:**
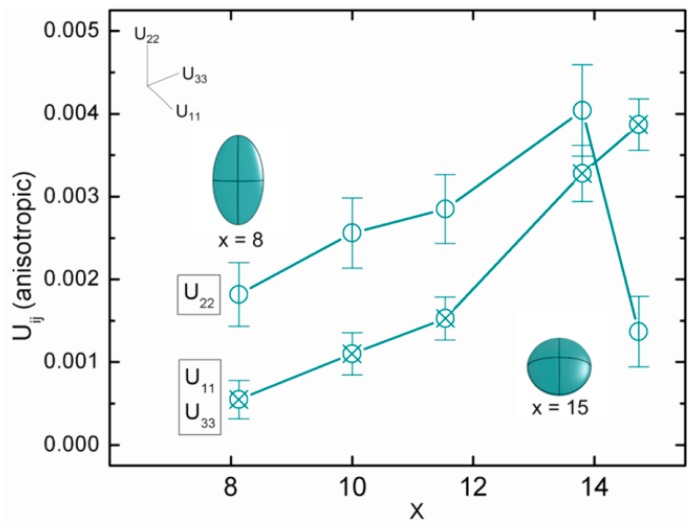
Anisotropic atomic displacement parameters (U_11_, U_22_, and U_33_) of the 6*d* site vs. composition *x* of Ba_8_Al_x_Si_46-x_ at 35 K. Reprinted with permission from Roudebush, J.H.; de la Cruz, C.; Chakoumakos, B.C.; Kauzlarich, S.M. Neutron Diffraction Study of the Type I Clathrate Ba_8_Al_x_Si_46-x_: Site Occupancies, Cage Volumes, and the Interaction between the Guest and the Host Framework. *Inorg. Chem.* **2012**, *51*, 1805–1812 [6]. Copyright (2012) American Chemical Society.

Although this review is concerned with the alkali metal guest group 13 Si clathrate type I phases, it is informative to review the guest ADPs for Ba_8_Al_x_Si_46-x_, since the amount of Al can vary from x ~ 8–15. Neutron powder diffraction at 35 K of Ba_8_Al_x_Si_46-x_ showed that when Al composition increases from 8 to 15, the anisotropic ADPs behave differently as presented in Figure 4. In the Figure, U_11_ and U_33_ are the two thermal displacement parameters parallel to the six-membered ring in the E_24_ cage, and U_22_ is direction perpendicular to six-membered ring. As the Al composition increases and the cage volume increases as well, U_22_ changed from a value larger than U_11_, U_33_ to a value smaller than those two. As shown in Figure 4, the thermal ellipsoid changes critically when Al compositions increased [6]. However, compare the anisotropic ADPs of 6*d* site in the case of A_8_Ga_8_Si_38_ and A_8_Ga_8_Si_38_ (A = K, Rb, Cs): the alkali metals at 6*d* sites behave similarly with those of Ba_8_Al_15_Si_31_ instead of Ba_8_Al_8_Si_38_, with the larger U elongated in the directions parallel to the six-membered ring (U_11_, U_33_) [13,14].

Unlike Ba_8_Al_x_Si_46-x_, the composition for A_8_Al_8_Si_38_ appears to be fixed [14,37], although minor adjustments appear to be possible [10,38]. Therefore, in order to tune the electronics, the composition K_8-x_Ba_x_Al_8+x_Si_38-x_ was investigated using metal hydrides for the alkali and alkaline earth metals and Al/Si arc melted mixtures heated together. The nominal compositions of x = 1.0, 1.5, and 2.0 were investigated. The structures were investigated for each composition with synchrotron radiation at 295 and 100 K. The 2*a* site with the smaller volume polyhedron was mostly substituted by Ba^2+^ rather than K^+^ guest atoms, consistent with the smaller ionic radius of Ba^2+^ [37]. Similar to the anisotropic ADP described above for the guest atom at the 6*d* site, the guest atom in the 6*d* site shows a larger displacement than the direction perpendicular to the six-membered ring of the 24-vertex cage. This is also observed for the Zn-containing phases [39].

## 4. Properties

The band structures of A_8_Ga_8_Si_38_ were first calculated using density-functional theory [25,26] and determined to be indirect bandgap semiconductors with the calculated bandgaps increasing with increasing mass of A. K_8_E_8_Si_38_ for E = Al, Ga, In was also calculated and showed a slight increase in bandgap with decreasing mass of E. Overall, the gaps were calculated to be 0.45 eV for Na_8_Ga_8_Si_38_ to 0.89 for Cs_8_Ga_8_Si_38_ while Eg value of Si with the diamond structure is calculated to be 0.65 eV. Since the experimental value of Eg of Si is 1.12 eV, these calculated values are adjusted according, corresponding to 0.92 and 1.36 eV for Na_8_Ga_8_Si_38_ and Cs_8_Ga_8_Si_38_, respectively. The experimental determination was for K_8_Ga_8_Si_38_ and the optical bandgap was estimated to be indirect at 1.2 eV [12,40]. With a higher level of theory and site preferences of E similar to that found experimentally, the bandgap of K_8_Al_8_Si_38_ was found to be larger than 1 eV [11]. The experimental measurements of A_8_Ga_8_Si_38_ follow the predicted trend, but are larger than the original calculations [13]. The values of the bandgap for K_8_Al_8_Si_38_ and A_8_Ga_8_Si_38_ (A = K, Rb, Cs) were determined from surface photovoltage spectroscopy [41,42] indicated as n-type and are in the range of 1.1–1.4 eV [13]. Na_8_Al_8_Si_38_ indicated an optical bandgap of 0.64 eV [9].

There has been significant interest and recent progress in the measurement of thermoelectric properties of these clathrates. Theoretical calculations indicated that that the dimensionless figure of merit, zT, for optimally doped compounds can reach 0.5 at 600 K. Hole-doped Cs_8_Ga_8_Si_38_ is predicted to provide a zT of 0.75 at 900 K. The figure of merit is a dimensionless parameter that provides a way to distinguish a favorable material for high performance as it combines the following thermoelectric parameters: Seebeck coefficient, resistivity, and thermal conductivity for a specific absolute temperature. Table 3 shows the data reported at 300 K for comparison. The thermal conductivity in all cases is low and the Seebeck coefficients are reasonable, but the electrical resistivity is too high in most cases. In the example of K_8_Al_8_Si_38_, it is apparent from the reported results that the precise stoichiometry greatly affects the electrical resistivity, as can be seen in Table 3 with the various entries [13,14,37] and for the one example that is slightly off stoichiometry, K_8_Al_7_Si_39_ [38].

**Figure 5:**
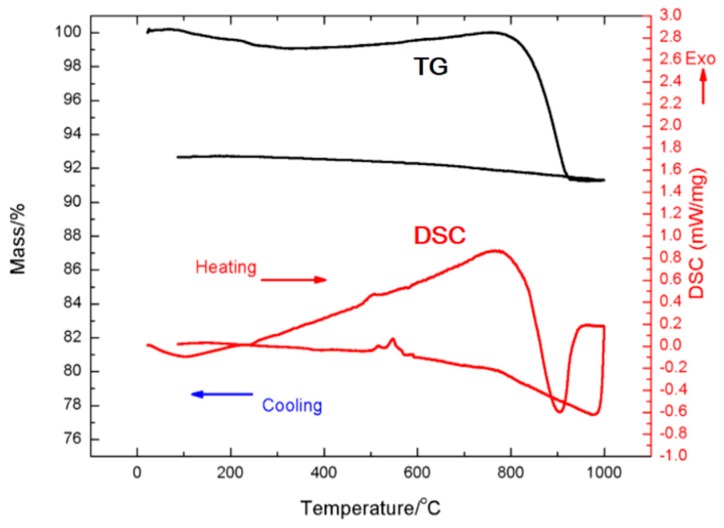
TG-DSC of K_8_Al_8_Si_38_ measured from 25 °C to 1000 °C heating and then cooling under flowing argon. Reprinted with permission from Sui, F.; Kauzlarich, S.M. Tuning Thermoelectric Properties of Type I Clathrate K_8-x_Ba_x_Al_8+x_Si_38-x_ through Barium Substitution. *Chem. Mater.* **2016**, *28*, 3099–3107 [37]. Copyright (2016) American Chemical Society.

Since the K_8_Al_8_Si_38_ phase showed the most promise with ease of synthesis, further optimization of that phase was considered. As indicated above, one method to optimize the transport properties is to focus on the Al-Si ratio and K_8_Al_7_Si_39_ shows the promise of this approach. Another method for decreasing the resistivity of the stoichiometric A_8_E_8_Si_38_ samples is to attempt to control the electron donation to the framework with mixed cations. The mixed alkali metal-alkaline earth Al-Si clathrate phases, K_8-x_Ba_x_Al_8+x_Si_38-x_ (x = 0, 1, 1.5, 2) were prepared and their thermoelectric properties measured. The K_8_Al_8_Si_38_ used in this study [37] had slightly different lattice parameters than those measured previously attributed to slightly different stoichiometry [13,14]. Thermogravimetric/differential scanning calorimetry (TG/DSC) measurements indicated that the K_8_Al_8_Si_38_ phase was stable up to 900 K (Figure 5), so temperature-dependent studies up to 900 K were performed on about 90% dense pressed pellets. The samples showed significantly lower electrical resistivities compared with stoichiometric A_8_E_8_Si_38_ samples.

**Figure 6:**
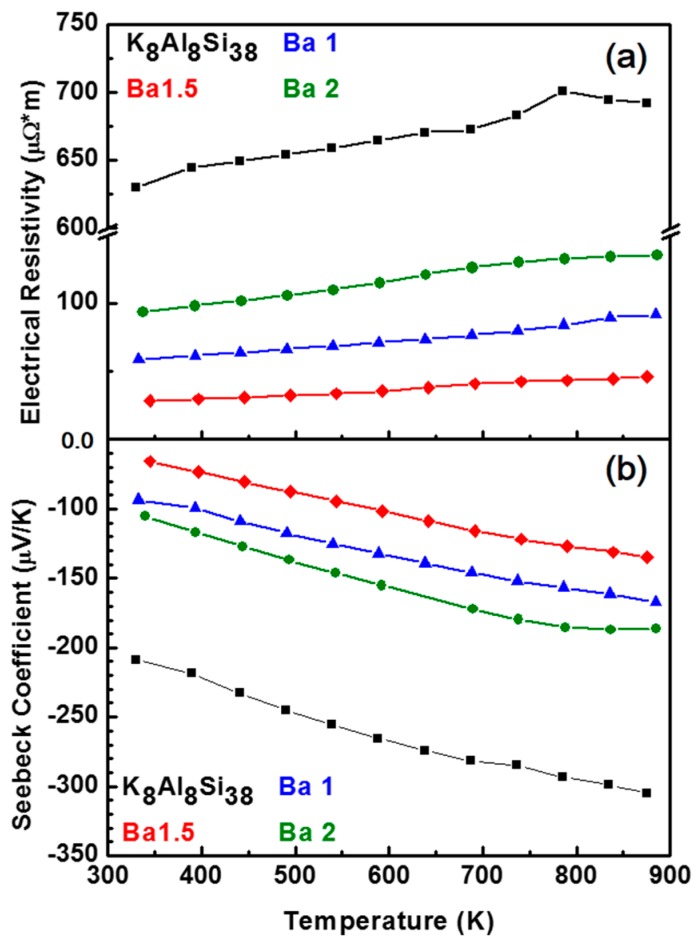
Temperature dependence of the (**a**) electrical resistivity and (**b**) absolute Seebeck coefficient measurements for K_8-x_Ba_x_Al_8+x_Si_38-x_ (x = 0, 1, 1.5, 2) samples from 323 K to 873 K. Reprinted with permission from Sui, F.; Kauzlarich, S.M. Tuning Thermoelectric Properties of Type I Clathrate K_8-x_Ba_x_Al_8+x_Si_38-x_ through Barium Substitution. *Chem. Mat.* **2016**, *28*, 3099–3107 [37]. Copyright (2016) American Chemical Society.

Preparing dense pellets of clathrates via spark plasma sintering is challenging, especially those with low melting points or potentially volatile guest atoms. In the case of K_8_Al_8_Si_38_, the samples decompose as K volatiles with the application of SPS. Therefore, while pellets with density of 80%–85% could be prepared for low temperature measurements (4 mm diameter pellets), high temperature spark plasma sintering of larger pellets provided samples with lower densities thereby making it difficult to obtain reliable experimental measurements of thermal conductivity. Pressed pellets of K_8-x_Ba_x_Al_8+x_Si_38-x_ (x = 0, 1, 1.5, 2) were prepared and the Seebeck coefficient and electrical resistivity measured (Figure 6) from room temperature to 1000 K.

**Figure 7:**
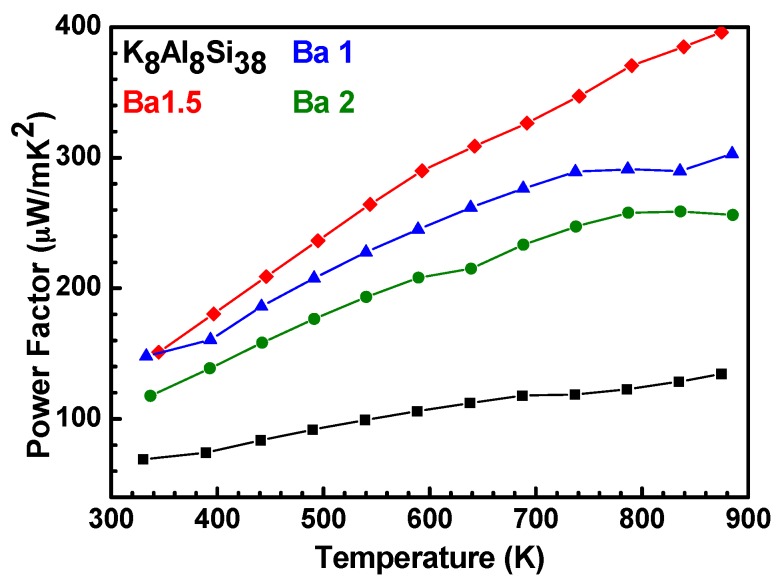
Thermoelectric power factors (S^2^σ) for K_8-x_Ba_x_Al_8+x_Si_38-x_ (x = 0, 1, 1.5, 2) samples from 323 K to 873 K. Adapted with permission from Sui, F.; Kauzlarich, S.M. Tuning Thermoelectric Properties of Type I Clathrate K_8-x_Ba_x_Al_8+x_Si_38-x_ through Barium Substitution. *Chem. Mat.* **2016**, *28*, 3099–3107 [37]. Copyright (2016) American Chemical Society.

Figure 6 shows that the K_8-x_Ba_x_Al_8+x_Si_38-x_ samples are all n-type with the lowest resistivity for x = 1.5. The resistivities do not correlate with x, as precise control over the Al-Si content is difficult to achieve with the hydride synthesis described in the manuscript [37]. The power factor for the same samples is provided in Figure 7, indicating that x = 1.5 is the best sample in the series.

If lattice thermal conductivities are extrapolated based on the low temperature measurements, and electronic thermal conductivities are calculated according to the Wiedemann-Franz relationship, the zT can be estimated at ~0.35 at 873 K, a significant value for an all earth abundant material, providing inspiration for those working on novel clathrates for high zT in a non-toxic, earth abundant material [37].

## 5. Synthesis

The simplest routes to the phases A_8_E_8_Si_38_ involve simply reacting the element in sealed Nb/Ta tubes at high temperatures. In particular, the gallium phases of A = K, Rb, Cs; E = Ga can be prepared in this manner. K_8_Ga_8_Si_38_ and Rb_8_Ga_8_Si_38_ were synthesized from on-stoichiometry reactions between pure elements in Nb/Ta tube heated to 1270 K and annealed at 970 K and their type I clathrate structure were reported [7,8]. K_7_B_7_Si_39_ can also be prepared from the elements, heating the mixtures in a Ta tube, encapsulated in fused silica, at about 1100 K. However, the reaction was not complete, and the impurities were removed by reaction with sodium hydroxide and concentrated acid [7,12]. To date, there has been little or no follow-up of the boron-containing phases.

**Figure 8:**
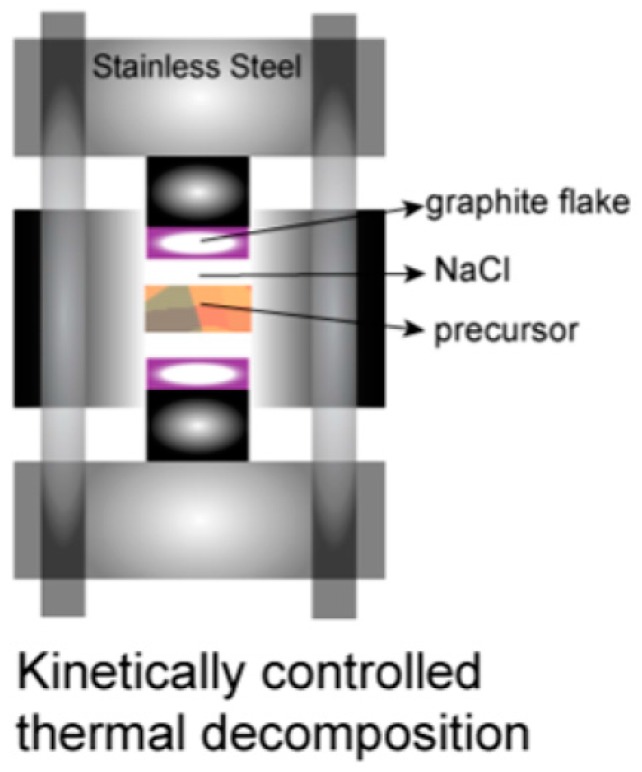
Schematic showing the kinetically controlled thermal decomposition (KCTD) set-up for the preparation of Na_8_Al_8_Si_38_. Reprinted with permission from Dong, Y.K.; Chai, P.; Beekman, M.; Zeng, X.Y.; Tritt, T.M.; Nolas, G.S. Precursor Routes to Complex Ternary Intermetallics: Single-Crystal and Microcrystalline Preparation of Clathrate-I Na_8_Al_8_Si_38_ from NaSi plus NaAlSi. *Inorg. Chem.* **2015**, *54*, 5316–5321 [9]. Copyright (2015) American Chemical Society.

Na_8_Al_8_Si_38_ can be synthesized in spark plasma sintering (SPS) and kinetically controlled thermal decomposition (KCTD) reactions with NaAlSi and NaSi mixture [9]. This route can lead to single crystals or phase-pure microcrystalline powders. Using different precursor rations, Na_8_Al_x_Si_46-x_ compositions were investigated, but while the diffraction peaks broadened, there was no direct evidence for a composition other than x ~ 8. Figure 8 shows a schematic of how the reaction is performed. The NaAlSi and NaSi mixture is placed in a custom-designed stainless steel apparatus under a uniaxial pressure of 20 MPa and heated for 9 h under dynamic vacuum (10^−6^ torr) at 953 K. During this reaction, the Na is removed in a chemically controlled manner from the NaAlSi/NaSi mixture via interaction with a graphite flake which is spatially separated from the mixture via NaCl. The NaCl stops a direct reaction of the Na with the graphite, so it is presumed to be a vapor-phase interaction of Na with the graphite flake. This procedure has been described in detail for the synthesis of Na-Si clathrate phases [43]. The product is carefully washed with ethanol and distilled water and dried in air. Caution should be used as NaSi can react violently with water.

High pressure synthesis of Na_8_Al_x_Si_46-x_ has also been investigated employing a mixture of NaSi, Al and Si as reagents, but only x~0.5 was prepared at 5.5 GPa and 1570 K [10]. This low x phase could not be produced as a phase-pure product and further synthetic efforts are necessary to provide a conclusion concerning whether x can be varied in this system. K_8_Al_8_Si_38_ was reported to be synthesized from KH and arc-melted Al/Si, sealed in a Ta tube jacketed in fused silica at 973 K. Ball milling KH and Al/Si together subsequent to heating enhanced the sample purity [13]. K_8-x_Ba_x_Al_8+x_Si_38-x_ could be prepared from ball milling BaH_2_ with KH and arc-melted Al/Si in the same manner as described above [37]. While this route provided fairly good control over the stoichiometry, caution should be taken when handling the metal hydrides that can react violently with water. A_8_Ga_8_Si_38_ (A = K, Rb, Cs) can be prepared from the pure elements reaction in a Ta tube jacketed in a fused silica ampoule at 973 K [13]. Excess alkali metals were used to obtain phase-pure samples and carefully removed with ethanol from samples. Caution when handling these samples is necessary as unreacted alkali metal can react violently with ethanol. Typically, the samples were initially allowed to oxidize slowly by exposing them over time to air, then the samples were covered with hexane to protect from water and ethanol slowly added before the mixture was sonicated. In the case of Cs_8_Cd_4_Sn_42_, phase-pure samples could be prepared by simple heating and annealing in a sealed metal tube [44]. It is possible that the Cd-containing Si or Ge phases can be prepared in a similar manner. Single-phase A_8_Al_8_Si_38_ (A = K, Rb, Cs) can also be prepared from Al and the respective alkali-metal halide salt flux, as reported by Baran et al. Similarly, Cs_8-x_Ga_8-y_Si_38+y_ was synthesized from a stoichiometric reaction of the elements in an alkali metal halide flux, while K_8_Zn_3.5_Si_42.5_ and Rb_7.9_Zn_3.6_Si_42.4_ were synthesized using a combined alkali metal halide/zinc flux [14,17]. Excess halides flux can be removed by dissolving in water as all of the clathrate phases are air stable. Unlike the Sn [45] or Ge clathrates [46], this is the only flux route reported to date for the synthesis of alkali metals containing group 13-silicon clathrates and, while it is reported to provide enough sample for pressed powders, it results in small crystals which are not sufficient for thermoelectric property measurements [14,17]. Therefore, there is still a significant challenge to grow large single crystals of good quality for property measurements of the Si clathrates with alkali metal guests.

## 6. Conclusions

New synthetic processes to prepare single-phase clathrates of all earth abundant and non-toxic elements have provided new clathrate type I phases that have potential for applications in energy storage and energy conversion. Metal hydrides, salt fluxes, and kinetically controlled decomposition of ternary phases provide methods for precise control over composition. All of the compositions discussed above show reasonable bandgaps for photovoltaics and low thermal conductivity, important to thermoelectrics. Controlling the carrier concentration by alkali metal-alkaline earth metal solid solutions resulted in a reasonable *zT*, suggesting that further improvements are possible. Controlling the guest atoms by means of solid solutions of K and Ba have been shown to be a productive way to systematically change the n-type carrier concentration. Further investigations of Si-Ge, Si-Sn, and more complex solid solutions (Al-Ga; Ga-In, for example) of these phases should be investigated with guidance from theory. The solid solutions of Zn(Cd)-E (E = Al, Ga, In) might provide a way to access hole-doped (p-type) clathrates. This is a rich area of chemistry, and progress towards other clathrate structures along with both guest and framework substitutions, provides a rich playground for new materials with technologically relevant properties.

## Figures and Tables

**Table 1:** Experimental Unit Cell Parameter of Type I Clathrate Phase A_8_E_8_Si_39_ (A = Na, K, Rb, Cs; E = Al, Ga).

Compound	a (Å)		Reference
	Powder (293 K)	Single Crystal (T)	
Na_8_Al_8_Si_38_	10.3260(1)	10.3170(1) (100 K)	[9]
K_8_Al_8_Si_38_	10.48072(2) ^‡^	10.4802(16) (90 K)	[11]
Rb_8_Al_8_Si_38_	10.53069(1)	10.517(1) (298 K)	[14]
Cs_8_Al_8_Si_38_	10.5848(4)	10.569(1) (298 K)	[14]
Na_8_Ga_8_Si_38_	-	-	-
K_8_Ga_8_Si_38_	10.424916(10) ^‡^	10.4270(3) (200 K)	[13]
Rb_8_Ga_8_Si_38_	10.470174(13)	10.4433(15) (200 K)	[13]
Cs_8_Ga_8_Si_38_	10.535069(15)	10.5339(3) (200 K)	[13]

^‡^ Synchrotron data.

**Table 2:** Comparison of Al and Ga’s site occupancy fraction (*s.o.f.*) at the Si framework sites, 6*c*, 24*k*, 16*i* of A_8_Tr_8_Si_38_ (A = K, Rb, Cs; Tr = Al, Ga) compounds.

Compounds	Atom	*s.o.f.* at 6*c*	*s.o.f.* at 24*k*	*s.o.f.* at 16*i*	Reference
K_8_Al_8_Si_38_	Al	75(3)%	12(1)%	4(1)%	Neutron powder diffraction [14]
Rb_8_Al_8_Si_38_	Al	60(4)%	15(2)%	5(3)%	
Cs_8_Al_8_Si_38_	Al	41(3)%	16(1)%	10(2)%	
K_8_Ga_8_Si_38_	Ga	61.0(7)%	17.7(4)%	3.2(3)%	Single crystal XRD [13]
Rb_8_Ga_8_Si_38_	Ga	52.0(9)%	17.8(4)%	0.4(4)%	
Cs_8_Ga_8_Si_38_	Ga	46.2(5)%	20.2(3)%	1.07(3)%	

**Table 3:** Experimental Seebeck coefficient, thermal conductivity, electrical resistivity and figure of merit zT for A_8_E_8_Si_38_ and related clathrates estimated at 300 K.

Phase	ρ (μΩ·m)	S $(\mu VK^{-1})$	κ $(WK^{-1}m^{-1})$	zT	Reference
Na_8_Al_8_Si_38_	4.3 × 10^3^	−22	2.03	1.7 × 10^−5^	[9]
K_8_Al_8_Si_38_	6.4 × 10^3^	−222	1.65	1.4 × 10^−3^	[14]
	1 × 10^3^	−90	1.80	1.4 × 10^−3^	[13]
	625	−210	1.80	1.2 × 10^−2^	[37]
K_8_Al_7_Si_39_	78.5	−65.5	1.7	9.6 × 10^−3^	[38]
Rb_8_Al_8_Si_38_	3.2 × 10^4^	−252	1.24	4.8 × 10^−4^	[14]
Cs_8_Al_8_Si_38_	5.4 × 10^6^	−300	0.93	5.3 × 10^−6^	[14]
K_8_Ga_8_Si_38_	2.0 × 10^3^	−25	0.51	1.8 × 10^−4^	[13]
Rb_8_Ga_8_Si_38_	1.0 × 10^4^	17	1.1	8.0 × 10^−6^	[13]
Cs_8_Ga_8_Si_38_	1.2 × 10^5^	30	1.7	1.3 × 10^−6^	[13]

## References

[1] Nolas G.S. (2014). The Physics and Chemistry of Inorganic Clathrates.

[2] Kauzlarich S.M., Sui F., Nolas G.S. (2014). Light Element Group 13–14 Clathrate Phases. The Physics and Chemistry of Inorganic Clathrates.

[3] Kasper J.S., Hagenmul P., Pouchard M., Cros C. (1965). Clathrate Structure of Silicon Na8Si46 and NaxSi136 (x <11). Science.

[4] Ramachandran G.K., McMillan P.F. (2000). K(7.62(1))Si(46) and Rb(6.15(2))Si(46): Two structure I clathrates with fully occupied framework sites. J. Sol. State Chem..

[5] Wosylus A., Veremchuk I., Schnelle W., Baitinger M., Schwarz U., Grin Y. (2009). Cs(8-x)Si(46): A type-I clathrate with expanded silicon framework. Chem. Eur. J..

[6] Roudebush J.H., de la Cruz C., Chakoumakos B.C., Kauzlarich S.M. (2012). Neutron Diffraction Study of the Type I Clathrate Ba_8_Al_x_Si_46-x_: Site Occupancies, Cage Volumes, and the Interaction between the Guest and the Host Framework. Inorg. Chem..

[7] Kroner R., Peters K., von Schnering H.G., Nesper R. (1998). Crystal structure of the clathrates K8Ga8Si38 and K8Ga8Sn38. Z. Krist. New Cryst. St..

[8] Von Schnering H.G., Kroner R., Menke H., Peters K., Nesper R. (1998). Crystal structure of the clathrates Rb8Ga8Sn38, Rb8Ga8Ge38 and Rb8Ga8Si38. Z. Krist. New Cryst. St..

[9] Dong Y.K., Chai P., Beekman M., Zeng X.Y., Tritt T.M., Nolas G.S. (2015). Precursor Routes to Complex Ternary Intermetallics: Single-Crystal and Microcrystalline Preparation of Clathrate-I Na_8_Al_8_Si_38_ from NaSi plus NaAlSi. Inorg. Chem..

[10] Imai M., Singh S.K., Nishio M., Yamada T., Yamane H. (2015). Synthesis of ternary Si clathrates in the A-Al-Si (A = Na and K) system. Jpn. J. Appl. Phys..

[11] He Y., Sui F., Kauzlarich S.M., Galli G. (2014). Si-based Earth abundant clathrates for solar energy conversion. Energy Environ. Sci..

[12] Imai M., Sato A., Udono H., Imai Y., Tajima H. (2011). Semiconducting behavior of type-I Si clathrate K8Ga8Si38. Dalton Trans..

[13] Sui F., He H., Bobev S., Zhao J., Osterloh F.E., Kauzlarich S.M. (2015). Synthesis, Structure, Thermoelectric Properties, and Band Gaps of Alkali Metal Containing Type I Clathrates: A(8)Ga(8)Si(38) (A = K, Rb, Cs) and K8Al8Si38. Chem. Mater..

[14] Baran V., Senyshyn A., Karttunen A.J., Fischer A., Scherer W., Raudaschl-Sieber G., Fässler T.F. (2014). A Combined Metal–Halide/Metal Flux Synthetic Route towards Type-I Clathrates: Crystal Structures and Thermoelectric Properties of A8Al8Si38 (A = K, Rb, and Cs). Chem. Eur. J..

[15] Jung W., Lörincz J., Ramlau R., Borrmann H., Prots Y., Haarmann F., Schnelle W., Burkhardt U., Baitinger M., Grin Y. (2007). K7B7Si39, a borosilicide with the clathrate I structure. Angew. Chem. Int. Ed. Engl..

[16] Condron C.L., Kauzlarich S.M., Ikeda T., Snyder G.J., Haarmann F., Jeglic P. (2008). Synthesis, Structure, and High-Temperature Thermoelectric Properties of Boron-Doped Ba8Al14Si31 Clathrate I Phases. Inorg. Chem..

[17] Baran V., Fässler T.F. (2015). Si-based Clathrates with Partial Substitution by Zn and Ga: K8Zn3.5Si42.5, Rb7.9Zn3.6Si42.4, and Cs8–xGa8–ySi38+y. Z. Anorg. Allg. Chem..

[18] Stefanoski S., Dong Y., Nolas G.S. (2013). Structural characterization and low-temperature physical properties of p-type single-crystal K(8)Ga(8.5)Sn(37.5) grown by self-flux method. J. Solid State Chem..

[19] Schäfer M.C., Bobev S. (2014). New Type-I and Type-II Clathrates in the Systems Cs–Na–Ga–Si, Rb–Na–Ga–Si, and Rb–Na–Zn–Si. Inorganics.

[20] Wei K.Y., Dong Y., Nolas G.S. (2016). Precursor routes to quaternary intermetallics: Synthesis, crystal structure, and physical properties of clathrate-II Cs8Na16Al24Si112. J. Solid State Chem..

[21] Nakamura K., Yamada S., Ohnuma T. (2013). Energetic Stability and Thermoelectric Property of Alkali-Metal-Encapsulated Type-I Silicon-Clathrate from First-Principles Calculation. Mater. Trans. JIM.

[22] He Y., Galli G. (2014). Nanostructured Clathrate Phonon Glasses: Beyond the Rattling Concept. Nano Lett..

[23] Takabatake T., Suekuni K., Nakayama T., Kaneshita E. (2014). Phonon-glass electron-crystal thermoelectric clathrates: Experiments and theory. Rev. Mod. Phys..

[24] Slack G.A., Rowe D.M. (1995). CRC Handbook of Thermoelectrics.

[25] Imai Y., Imai M. (2011). Chemical trends of the band gaps of idealized crystal of semiconducting silicon clathrates, M(8)Si(38)A(8) (M = Na, K, Rb, Cs; A = Ga, Al, In), predicted by first-principle pseudopotential calculations. J. Alloys Compd..

[26] Imai Y., Watanabe A. (2011). Chemical Trends of the Band Gaps in Semiconducting Silicon Clathrates. Phys. Procedia.

[27] Christensen M., Johnsen S., Iversen B.B. (2010). Thermoelectric clathrates of type I. Dalton Trans..

[28] Ramachandran G.K., Dong J.J., Diefenbacher J., Gryko J., Marzke R.F., Sankey O.F., McMillan P.F. (1999). Synthesis and X-ray characterization of silicon clathrates. J. Sol. State Chem..

[29] Shimizu H., Imai T., Kume T., Sasaki S., Kaltzoglou A., Fässler T.F. (2008). Raman spectroscopy study of type-I clathrates A(8)Sn(44)square(2) (A = Rb, Cs) and Rb8Hg4Sn42. Chem. Phys. Lett..

[30] Veremchuk I., Wosylus A., Boehme B., Baitinger M., Borrmann H., Prots Y., Burkhardt U., Schwarz U., Grin Y. (2011). Preparation and Crystal Structure of the Clathrate-I Cs8-xGe44+y square(2-y). Z. Anorg. Allg. Chem..

[31] Zhao J.-T., Corbett J.D. (1994). Zintl Phases in Alkali–Metal–Tin Systems: K8Sn25 With Condensed Pentagonal Dodecahedra of Tin. Two A8Sn44 Phases With a Defect Clathrate Structure. Inorg. Chem..

[32] Dubois F., Fässler T.F. (2005). Ordering of Vacancies in Type-I Tin Clathrate: Superstructure of Rb8Sn44[]2. J. Am. Chem. Soc..

[33] Beekman M., Nolas G.S. (2007). Transport Properties of the Binary Type I Clathrate K8Ge44[]2. Int. J. Appl. Ceram. Technol..

[34] Bobnar M., Böhme B., Wedel M., Burkhardt U., Ormeci A., Prots Y., Drathen C., Liang Y., Nguyen H.D., Baitingera M. (2015). Distribution of Al atoms in the clathrate-I phase Ba8AlxSi46−x at x = 6.9. Dalton Trans..

[35] Witte J., Schnering H.G. (1964). Die Kristallstruktur von NaSi and NaGe. Z. Anorg. Allg. Chem..

[36] Westerhaus W., Schuster H.U. (1979). Darstellung and Struktur von NaAlSi and NaAlGe. Z. Naturforsch B.

[37] Sui F., Kauzlarich S.M. (2016). Tuning Thermoelectric Properties of Type I Clathrate K_8-x_Ba_x_Al_8+x_Si_38-x_ through Barium Substitution. Chem. Mat..

[38] Singh S.K., Mochiku T., Ibuka S., Isoda Y., Hoshikawa A., Ishigaki T., Imai M. (2015). Synthesis and transport properties of ternary type-I Si clathrate K8Al7Si39. Jpn. J. Appl. Phys..

[39] Baran V., Fischer A., Scherer W., Fässler T.F. (2013). Synthesis of Large Single Crystals and Thermoelectrical Properties of the Type-I Clathrate K8Zn4Sn42. Z. Anorg. Allg. Chem..

[40] Iioka M., Udono H., Imai M., Aoki M. (2015). Band Structure Characterization of K8Ga8Si38 Clathrates by Optical Measurement. Jpn. J. Appl. Phys. Conf. Proc..

[41] Kronik L., Shapira Y. (1999). Surface photovoltage phenomena: Theory, experiment, and applications. Surf. Sci. Rep..

[42] Kronik L., Shapira Y. (2001). Surface photovoltage spectroscopy of semiconductor structures: At the crossroads of physics, chemistry and electrical engineering. Surf. Interface Anal..

[43] Stefanoski S., Beekman M., Wong-Ng W., Zavalij P., Nolas G.S. (2011). Simple Approach for Selective Crystal Growth of Intermetallic Clathrates. Chem. Mater..

[44] Wilkinson A.P., Lind C., Young R.A., Shastri S.D., Lee P.L., Nolas G.S. (2002). Preparation, Transport Properties, and Structure Analysis by Resonant X-ray Scattering of the Type I Clathrate Cs8Cd4Sn42. Chem. Mater..

[45] Tanaka T., Onimaru T., Suekuni K., Mano S., Fukuoka H., Yamanaka S., Takabatake T. (2010). Interplay between thermoelectric and structural properties of type-I clathrate K_{8}Ga_{8}Sn_{38} single crystals. Phys. Rev. B.

[46] Toberer E.S., Christensen M., Iversen B.B., Snyder G.J. (2008). High temperature thermoelectric efficiency in Ba8Ga16Ge30. Phys. Rev. B.

